# Accelerated microevolution in an outer membrane protein (OMP) of the intracellular bacteria *Wolbachia*

**DOI:** 10.1186/1471-2148-10-48

**Published:** 2010-02-17

**Authors:** Laura Baldo, Christopher A Desjardins, Jacob A Russell, Julie K Stahlhut, John H Werren

**Affiliations:** 1 Department of Biology, University of California, Riverside, CA, USA; 2 Biodiversity Institute of Ontario, University of Guelph, Guelph, ON, N1G 2W1, Canada; 3 Department of Biology, University of Rochester, Rochester, NY, USA; 4 Department of Organismic and Evolutionary Biology, Harvard University, Cambridge, MA 02138, USA; 5 Current address: Zoology Institute, University of Basel, Vesalgasse 1, CH-4051 Basel, Switzerland

## Abstract

**Background:**

Outer membrane proteins (OMPs) of Gram-negative bacteria are key players in the biology of bacterial-host interactions. However, while considerable attention has been given to OMPs of vertebrate pathogens, relatively little is known about the role of these proteins in bacteria that primarily infect invertebrates. One such OMP is found in the intracellular bacteria *Wolbachia*, which are widespread symbionts of arthropods and filarial nematodes. Recent experimental studies have shown that the *Wolbachia *surface protein (WSP) can trigger host immune responses and control cell death programming in humans, suggesting a key role of WSP for establishment and persistence of the symbiosis in arthropods.

**Results:**

Here we performed an analysis of 515 unique alleles found in 831 *Wolbachia *isolates, to investigate WSP structure, microevolution and population genetics. WSP shows an eight-strand transmembrane β-barrel structure with four extracellular loops containing hypervariable regions (HVRs). A clustering approach based upon patterns of HVR haplotype diversity was used to group similar WSP sequences and to estimate the relative contribution of mutation and recombination during early stages of protein divergence. Results indicate that although point mutations generate most of the new protein haplotypes, recombination is a predominant force triggering diversity since the very first steps of protein evolution, causing at least 50% of the total amino acid variation observed in recently diverged proteins. Analysis of synonymous variants indicates that individual WSP protein types are subject to a very rapid turnover and that HVRs can accommodate a virtually unlimited repertoire of peptides. Overall distribution of WSP across hosts supports a non-random association of WSP with the host genus, although extensive horizontal transfer has occurred also in recent times.

**Conclusions:**

In OMPs of vertebrate pathogens, large recombination impact, positive selection, reduced structural and compositional constraints, and extensive lateral gene transfer are considered hallmarks of evolution in response to the adaptive immune system. However, *Wolbachia *do not infect vertebrates. Here we predict that the rapid turnover of WSP loop motifs could aid in evading or inhibiting the invertebrate innate immune response. Overall, these features identify WSP as a strong candidate for future studies of host-*Wolbachia *interactions that affect establishment and persistence of this widespread endosymbiosis.

## Background

Outer membrane proteins (OMPs) of pathogenic bacteria are widely recognized as crucially involved in bacterial interactions with eukaryotic hosts [1]. They have thus been the subject of extensive studies aimed to clarify how they evolve and whether their patterns of divergence are informative about the biology of host-bacteria dynamics. OMPs are involved in a large repertoire of functions, including bacterial invasion and defense, transportation of various molecules, adhesion and signaling pathways [1,2]. OMPs of mammalian pathogenic Proteobacteria often function as antigens [3], and several of these proteins are currently targets for vaccine development against important human pathogens, such as *Ehrlichia, Rickettsia, Haemophilus influenzae*, and *Neisseria meningitidis *[4,5].

OMPs are highly variable and among the fastest evolving microbial proteins [6,7]. Despite a large diversity of composition and function, they do share genetic and structural features that allow their identification as surface proteins, primarily via bioinformatic prediction [7]. OMPs show a characteristic transmembrane β-barrel structure, formed by an even number of antiparallel sheets, connected to loops of variable length at the extracellular side and to short turns containing both N and C termini at the periplasmic side. Given the key role of OMPs in the interactions with the host, a large number of studies have been devoted to uncover trends in the molecular evolution of these proteins [4,8-10]. Typically, residues in the β-barrel show the highest conservation, while variability mainly affects the conformational domains located in the extracellular loops, which can function as receptors and can be highly antigenic - e.g. P28 OMPs of *Ehrlichia*, Opa proteins of *Neisseria *and MSP2 proteins of *Anaplasma *[11-14].

While considerable attention has been given to OMPs of vertebrate pathogens, the role and evolution of OMPs in the establishment and persistence of both pathogenic and non-pathogenic microbial associations found in invertebrates remain largely unknown. Recent studies have shown that both the vertebrate and invertebrate immune systems can confer specific protection against bacterial infections and share comparable defensive solutions [15], making OMP of invertebrate pathogens important candidates for investigating host/symbiont interaction dynamics. In addition, for those vertebrate pathogens that are vectored by arthropods, selection by the invertebrate innate immune system could shape the virulence of vertebrate pathogens vectored by invertebrates [16]. Therefore, studies of OMPs in bacteria that infect invertebrates, but not vertebrates, could reveal to what extent selection in invertebrates shapes OMP diversity and evolution.

The *Wolbachia *surface protein (WSP) is an OMP found in the intracellular bacteria of the genus *Wolbachia *[17], a very widespread and important group of endosymbionts of arthropods and filarial nematodes. Current estimates indicate that around 60% of arthropod species worldwide are infected with this intracellular bacterium [18]. *Wolbachia *belong to the Rickettsiales and relatives are important vertebrate pathogens within the genera *Rickettsia, Ehrlichia *and *Anaplasma *[19]. However, in contrast to some notable members of these related genera, *Wolbachia *are not pathogens of vertebrates. Instead, theyare mostly known to be "reproductive parasites" of arthropods [20-22], while in filarial worms and some insects they are required for their hosts' survival and provide them with some benefits [23-25].

The function of WSP in *Wolbachia *remains unknown, although several lines of evidence suggest that it may be an important mediator of the host/symbiont interaction. First, WSP is a dominant protein constituent of infected *Drosophila *eggs [17]. Experimental studies have shown that WSP can activate the innate immune response in humans via interaction with Toll-like receptors [26], and trigger a potent inflammatory response in both human and canine filariasis [27]. Recently, WSP has been shown to delay apoptosis in human polymorphonuclear cells (PMNs), typically involved in the innate immune response against microbial pathogens [28]. Finally, inoculation of WSP in BALB/mice induces the expression and production of nitric oxide, an important toxic component used by the immune response against bacteria [29]. The above studies indicate that WSP can induce host immune responses and recent hypothesis predicts WSP as an important player in the establishment and persistence of the symbiosis via apoptosis inhibition [30].

WSP shows a heterogeneous pattern of amino acid diversity characteristic of other OMPs, marked by four distinct hypervariable regions (HVRs) interspaced by conserved strings of amino acids (CRs) [31]. Variants at each HVR have been frequently exchanged across bacterial strains, generating highly chimeric proteins [31]. While shuffling of HVR motifs is apparent, the primary source of such remarkable amino acid diversity at HVRs remains unknown. Furthermore, it is unclear whether this genetic diversity is adaptive; because arthropod *Wolbachia *do not infect vertebrate hosts, selection acting on the protein is not due to a response to the vertebrate adaptive immunity (e.g. antibodies). Other forces, therefore, are likely to be shaping the evolution of this protein.

Here we investigated structure and molecular evolution of WSP using 515 distinct alleles found in 831 host isolates, representing the largest sequence dataset available to date for *Wolbachia*. The first an *in silico *prediction of the three-dimensional structure of WSP is presented. Using the predicted structure as framework, we investigated the microevolutionary forces that drive the early diversification of WSP proteins by means of clusters of closely related proteins based upon haplotype categories for individual HVRs. This approach eliminates the problems of alignment due to extensive divergence within the HVRs, and allows identification of closely related HVRs (in different protein variants), thus providing a ready classification of variation generated via point mutation versus recombination in recently divergent proteins. We found that WSP shows a rapid turnover of amino acid sequences via both high rates of recombination and positive selection typical of immune antigens under strong selection for diversification. These appear as hallmarks of an ongoing arms race between the host and *Wolbachia *and identify WSP as a strong candidate for future studies of host-*Wolbachia *interactions.

## Methods

### *In silico *prediction of WSP structure

Discrimination of WSP among globular, inner and outer membrane proteins was assessed based on a position specific scoring matrix (PSSM) profiles approach, implemented in TMBETADISC-RBF [32]. Prediction was confirmed by querying the complete WSP sequence from wMel to the HHomp database, available at http://toolkit.tuebingen.mpg.de/, which detects sequence homology to known outer membrane proteins (OMPs) based on sequence-profiles identified with Hidden Markov Models **(**HMMs). The WSP two and three-dimensional structures were then predicted using HHpred [33], which uses structurally related proteins as template. Results were inputted into MODELLER [34] using a multiple alignment for modeling of the tertiary structure. The three-dimensional model was visualized in cn3D version 4.1 [35]. HHpred has been shown to perform better over simpler approaches (BLAST and PSIBLAST) when template-target similarity is lower than 40% [36], as is the case with WSP and homologous proteins. To correct for diversity among WSP proteins, we also generated three-dimensional models using a diverse set of divergent WSP genotypes, besides WSP from wMel. WSP topology with respect to the outer membrane lipid bilayer was predicted by the posterior decoding method using PRED-TMBB, software based on Hidden Markov model [37]. Analysis of the hydrophobic and hydrophilic indexes was performed using the Kyte and Doolittle scale [38].

### Sequence Mining

The nucleotide sequences were either generated during this study or retrieved from Genbank. New sequences were obtained using standard primers and protocols available at http://pubmlst.org/wolbachia/wsp/info/protocol.shtml. The procedure for Genbank retrieval was as follows: all nucleotide *wsp *sequences present in Genbank were downloaded and redundancy discarded. The set of unique *wsp *sequences that met the length requirement to be assigned to an allele (see below) and that showed no ambiguous sites were retained. Together with the sequences generated in this study a total of 515 distinct alleles were obtained. All sequences were then compared against the NCBI nucleotide database with BLASTN. For all exact matches (100% identity and coverage), all available host taxon and country of origin information were collected. Multiple entries having the same host species and same *wsp *allele were retained if they differed by locality information. Several alleles were sequenced during this study and their host information was combined with information collected from Genbank. Overall, 831 distinct records were collected (see additional file 1).

### Sequence Typing

**Figure 1:**
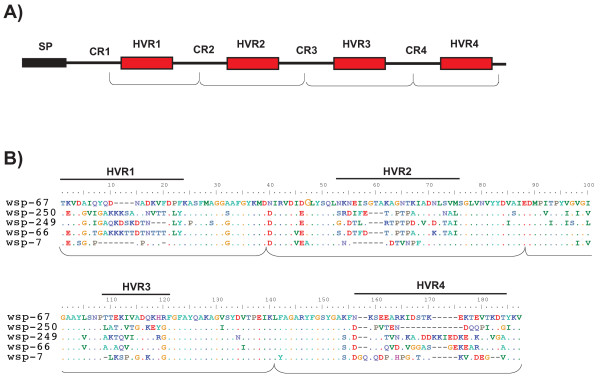
**WSP gene structure**. (A) Schematic representation of WSP structure depicting the signal peptide (SP), the four hypervariable regions (HVRs), interspaced by four conserved regions (CRs). (B) Representatives of most diverse WSP sequences named by a corresponding allele. Sections used for typing include HVR motifs plus short stretches of the two flanking CRs (bracketed below the two alignments).

All 515 alleles were characterized according to the *wsp *typing system previously developed [39]. Briefly, each unique nucleotide sequence within a defined range of nucleotides was assigned an allele number and translated into amino acids. The amino acid sequence was partitioned in four consecutive peptides, each encompassing one of the four HVRs and a short portion of the two flanking conserved regions. For convenience here we refer to each of these four sections as HVR (see Fig. 1A for a schematic representation). Each unique HVR peptide was assigned a number, thus a protein haplotype is identified by a profile of four peptide numbers (i.e. a WSP profile). Each *wsp *allele is always associated with a single WSP profile; however, the same WSP profile can be associated to different alleles (i.e. synonymous alleles). This typing method was developed to overcome the difficulty of managing *wsp *nucleotide sequences, which are highly variable in length and show extremely low homology at the four HVRs, thus making alignment reconstructions impracticable (see example in Fig. 1B). Moreover, the four HVRs of *wsp *undergo extensive recombination, which results in mixing HVRs peptides among proteins [31]. Such a typing system, therefore, provides a useful means for spotting recombination events involving divergent HVRs among otherwise closely related WSP sequences (see also below). All 515 alleles and corresponding HVR haplotype profiles were uploaded into the *wsp *database [40].

Analysis of nucleotide genetic diversity and GC content was performed using DNAsp version 4.50 [41,42]. GC content at the third (synonymous only) position was calculated for each of the six largest WSP complexes (C1-C6, see below for complex definition) and for the five MLST genes. Amino acid divergence was calculated in PAUP version 4.0b10 [43].

To explore whether additional intragenomic sources might contribute to *wsp *variability (such as pseudogenes or other noncoding sequences), complete *wsp *and single HVR nucleotide sequences from *Drosophila melanogaster *and *Culex pipiens *were BLASTN against the *Wolbachia *genomes from these two host species.

### Identification of protein complexes

We identified complexes of closely related WSP proteins using the clustering program eBURST V3 [44] and employing the matrix of WSP profiles to depict evolutionary relationships among WSP genotypes. This approach is superior to conventional phylogenetic analyses, in this case, because it reveals localized recombination (i.e. involving short intragenic sequences) among otherwise similar WSP sequences, without incorrectly inferring such sequences as phylogenetically distant. The program eBURST is typically used for analysis of MLST allelic profiles (where each allele at each MLST locus is given a number and an allelic profile is the combination of allele numbers at the MLST loci) and assumes an epidemic model of population structure for building the clusters: that is, it assumes that a founder genotype (an allelic profile) initially rises in frequency and subsequently diversifies to produce minor variants (i.e. single locus variants, SLV), hence producing a "clonal complex" of closely related genotypes. Given a matrix of allelic profiles, eBURST predicts the ancestral genotype to be the profile with the greatest number of single locus variants. Often the founder is also the most frequent profile in a complex (in terms of isolates in which is found).

Here we applied the same clustering algorithm to a single locus, thus using WSP profiles. WSP profiles were clustered into "WSP complexes", where a WSP complex is a group of related WSP profiles that differ at a single HVR peptide (here named single HVR variant, SHV) with respect to the ancestral profile. The ancestral profile of each complex is predicted by eBURST to be the WSP profile with the highest number of SHVs. Profiles that could not be assigned to a group were named singletons.

### Recombination versus mutation estimates

We used the complexes identified by eBURST to estimate the relative contribution of recombination versus mutation that give rise to new proteins. Within complexes, mutant profiles were discriminated from recombinant profiles (and associated alleles) by assessing whether SHVs diverged by mutation or recombination. The procedure was as follows. First, within each WSP complex, all WSP profiles were compared to the ancestral profile and the number of amino acid changes at their SHV was annotated. In case of complexes of two profiles we performed a simple pairwise comparison. This allowed a first screening for major recombinant proteins, defined as the WSP profiles that showed 4 or more amino acid polymorphic sites at their SHVs (including both amino acid substitutions and indels) with respect to the ancestral type. All other profiles within a complex, which then showed 1 to 3 polymorphic sites at their SHVs, were compared to the whole dataset and nucleotide substitution patterns inspected. If a SHV peptide was shared among profiles in different complexes, then mutation was assumed when the nucleotide substitution patterns at their SHV differed. In cases of matching substitution patterns, a) a single nucleotide substitution (thus nonsynonymous) was assumed to be arisen by convergence and the profiles retained as mutants, b) multiple shared nucleotide substitutions were considered sign of recombination and the recombinant profile was predicted to be the one with highest number of amino acid changes with respect to its ancestral profile. If equal, both profiles were labeled as recombinants. The relative contribution of recombination to mutation in generating protein diversity was measured as a ratio of recombination to mutation per protein and per HVR. We are aware that this approximation does not take into account mutation and recombination events that did not result in amino acid changes (i.e. synonymous substitutions); indeed our goal was to explore the contribution of the two forces in promoting protein diversity, and not synonymous allele diversity. In addition, we have also examined recombination occurrence among distinct alleles carrying only synonymous substitutions (see below). The recombination analyses were also expanded to profiles members of subgroups and subgroup founders (coded as double HVR variants, DHVs).

### Prediction of the ancestral alleles

Ancestral proteins predicted by eBURST can be coded by multiple alleles within a complex. To identify the ancestral allele and confirm eBURST prediction we proceeded as follows. First, within a complex, all alleles associated to mutant profiles, including synonymous alleles, were further analyzed at the nucleotide level to exclude recombination occurrence using the method of Betran et al [45], implemented in the DNAsp. Second, the ancestral allele of each complex was predicted using a statistical parsimony method [46] carried out in TCS software version 1.21 [47].

### Analysis of selective pressures

Selection on mutant alleles was investigated using the complexes previously detected. For complexes that included at least four sequences (n = 25, including also those coding for DHVs) all alleles were tested for neutrality, using the Tajima's D [48]. For complexes of three or more alleles for which the ancestral allele was identified (n = 29), rates of non-synonymous substitutions (dN) and synonymous substitutions (dS) per codon were estimated using the codeml program in PAML package [49,50]. Specifically, for each complex we generated a simple star tree with a bifurcation at the deepest node to assign the root (corresponding to the ancestral allele) and used it as input file for codeml. Four models were tested: the nearly neutral model (M1), which assumes a proportion *p*_0 _of conserved sites with *ω*_0 _< 1 and *p*_1 _= 1 - *p*_0 _of neutral sites with *ω *= 1; the positive-selection model (M2), which includes an additional class of sites with frequency *p*_2 _= 1 - *p*_0 _- *p*_1 _and with *ω*_2 _estimated from the data; the model M7, which assumes that *ω *is β-distributed and provides a flexible null hypothesis for testing positive selection; and the model M8, which includes one additional *ω *class of sites with respect to M7, estimated from the data. We performed two likelihood ratio tests (LRTs) comparing likelihood scores of M1 vs M2 and M7 vs M8. The presence of positively selected sites within each complex was determined by concordance between the two best-selected models using the Bayes Empirical Bayes analysis (BEB).

To explore selective pressures acting on partitions of *wsp*, the nucleotide alignment including the 515 alleles was divided into seven sections corresponding to hvr1, CR2, hvr2, CR3, hvr3, CR4 and hvr4. For this particular analysis, each hvr (here non capitalized to distinguish them from sections used for typing) comprises only the strings of hypervariable amino acids, thus excluding any portions of the flanking conserved regions. Section boundaries for hvrs were based on the alignment in Baldo *et al*. [31], where a similar analysis was performed using a much smaller dataset. Within each of the seven sections, redundancy of nucleotide sequences was discarded and sequences were grouped using BlastClust, available at http://toolkit.tuebingen.mpg.de/, based on 95% cutoff of nucleotide identity and 100% matching length. This clustering approach considers related regions of *wsp *even if inserted in a recombinant background. For each *wsp *section, dN/dS ratios were estimated at each cluster of three or more sequences using the method of Nei and Gojobori (1986) [51], implemented in DNAsp. Average values across groups within each section were then calculated. For few groups within hvrs, dN/dS values were not available, as sequences diverged only by nonsynonymous substitutions returning no ratios; although suggestive of positive selection, these groups were conventionally assigned dN/dS = 1 as a conservative estimate.

### Analysis of synonymous allele variants

For each of the 435 proteins in our dataset, number of allele variants, average synonymous diversity and number and type of polymorphisms were estimated using DNAsp. As a control, we compared these estimates to those obtained for the five MLST housekeeping genes, using a dataset of published and partly unpublished data. Statistical significance of difference in values between *wsp *and each of the five MLST genes was inferred performing a Wilcoxon two-sample test.

### Statistical association of WSP sequences with host taxa

The rarefaction curve was built using the online calculator available at http://www2.biology.ualberta.ca/jbrzusto/rarefact.php. Curve fitting to predict the asymptotic number of WSP proteins was performed using the on-line regression analysis at site http://www.xuru.org/rt/NLR.asp#Manually. The 37 points along the curve were used for curve fitting to formulae allowing for three parameters.

We tested whether genetic distances between WSP protein sequences that are found in hosts within the same genus were more similar than they would be by random chance. We choose to analyze proteins instead of nucleotide sequences to facilitate generation of alignments and because WSP amino acid diversity largely reflects the nucleotide diversity. The procedure was as follows: the 515 *wsp *sequences were translated into amino acids, aligned using ClustalX [52] and manually curated in Bioedit vs7.0.4.1 [53]. The HVR4 was eliminated from the analyses as difficult to align and highly recombinant. A distance matrix was generated based on the amino acid alignment using PAUP version 4.0b10 [43].

To determine whether WSP sequences were significantly associated with host genera, we sub-sampled the initial dataset to comprise only one host species per *wsp *allele, that is, the dataset included multiple representatives of the same species only in case they carried distinct *wsp *alleles. This avoids overrepresentation of a single *wsp *sequence found in the same host species but from different geographical regions. The final dataset included 732 entries. In order to create a null distribution, we generated 1000 pseudoreplicates by randomly resampling host taxa without replacement across all WSP sequences. For each pseudoreplicate we calculated the mean and median pairwise distances between WSP sequences within the taxonomic group being tested. We then compared the original mean and median values with the resulting pseudoreplicate distribution in a one-tailed test to determine p-values. For this association study we first performed a global analysis resampling all WSP pairwise distances within and between host genera separately and averaging the two sets of values; we then tested the association within individual genera.

## Results

### Three-dimensional structure prediction of WSP: an eight β-barrel OMP

**Figure 2:**
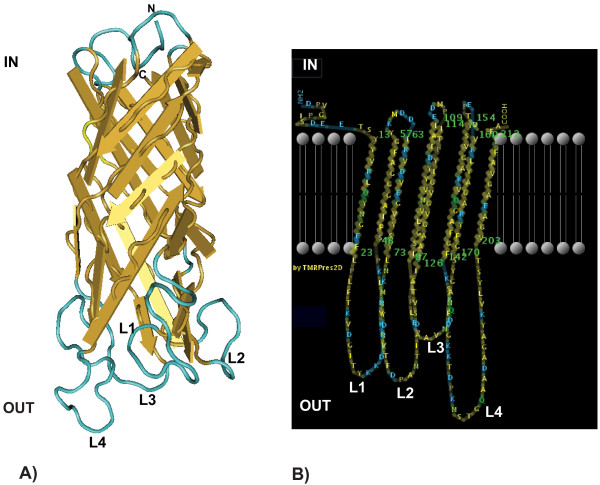
**Predicted folding and localization of WSP in the outer membrane**. WSP shows an eight antiparallel stranded β-barrel structure with four highly hydrophilic loops (L1-4) protruding into the extracellular side (OUT). C- and N-termini are in the periplasmic side (IN). Loop size and amino acid content greatly vary among WSP sequences, thus exact folding and localization for these extracellular regions cannot be reliably predicted.

WSP was identified as an OMP by the PSSM profiles approach and by BLAST search against the HHomp database (P = 100%). The best BLAST match outside *Wolbachia *was the major outer membrane protein P28-14 of *Ehrlichia muris *(AccNo. ABD93654, 29% identity, 43% of positives, 20% gaps), and second best match was the surface antigen MSP4 of *Ehrlichia canis *str. Jake (YP_303460, 29% identity, 43% of positives, 22% gaps). Among proteins with characterized three-dimensional structure, HHpred search identified the outer membrane protein NspA from *Neisseria meningitidis *as most resembling WSP (23% identity, P = 99.96, E-value = 8.7e-27). Use of a set of divergent WSP sequences also returned the same best hits. Based on a concordance of structure prediction between the two programs, WSP of the wMel strain was then modeled based on NspA. Results of the modeling showed an eight β-barrel structure with four extracellular loops (Fig. 2A). Although overall identity between WSP and NspA is only 23%, much of the variability between the two proteins is associated to the hypervariable loops, much longer in WSP, while secondary structure and amino acid strings interspacing the hypervariable loops are quite conserved. In general, hypervariable loops are not conserved among predicted eight β-barrel OMPs or among WSP sequences, signifying that loops do not greatly contribute to the folding and stability of the protein.

The analysis of the hydrophilic and hydrophobic pattern (Kyte & Doolittle scale) and prediction of WSP position on the lipid bilayer (Fig. 2B) confirmed that WSP shows a typical eight B-barrel structure with four hydrophilic extracellular loops and periplasmic turns connected to a β-barrel core containing predominantly the neutral or polar residues valine, alanine, glycine and tyrosine, in accordance to the typical composition of β-barrel proteins [7].

This computationally based structural analysis provided a framework to compare the patterns of protein evolution in different predicted functional domains.

### Genetic diversity of WSP

Based on the 515 alleles analyzed, WSP proteins can differ by as much as 43% of their amino acid content and 13% in length (Table 1). This variability is largely associated to the HVR motifs, contained in the loops, which show little homology and are rich in indels (see Fig. 1B for an example). In particular, HVR4 sequences (located in L4) can differ by up to 42% in length and are thus responsible for a large part of this protein diversity. The number of distinct peptides is similar across the four HVRs, ranging from 182 to 207 (Table 1). In contrast, the short regions interspacing HVRs (CRs) show an average amino acid homology >90% and no indels, likely due to structural constraints. The CRs largely form the β-barrel scaffold in the predicted three-dimensional structure, suggesting strong sequence constraints on this structure.

**Table 1:** WSP protein and single HVR peptide genetic diversity based on 515 alleles

	Amino acid length (range)	No. of prot or pept
WSP*	155-179	435
**HVR1**	30-38	182
**HVR2**	44-50	207
**HVR3**	50-52	207
**HVR4**	26-45	202

*Whole protein, excluding the first 51-2 amino acids of the 5' end and the last 15 amino acids of the 3'end.

Because WSP sequences show very low homology across the diversity within their individual HVRs, global phylogenetic reconstructions or a distance-related approach for studying WSP evolution is not practical. To circumvent the problem of such extensive variation among proteins and HVRs, we focused on recently diverged proteins that still retain true homology. This approach allowed us to identify recent events of recombination and mutation in evolving proteins.

### Clusters of closely related WSP

**Figure 3:**
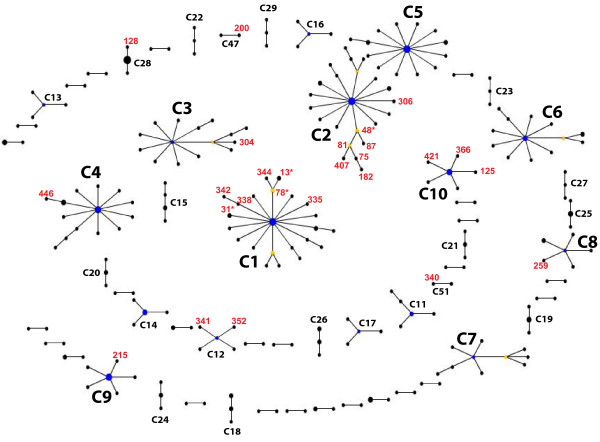
**Clusters of closely related WSP proteins (n = 252) estimated by eBURST**. Circles correspond to distinct proteins; area of the circles is proportional to the number of allele variants coding for a protein. Clusters of linked proteins (i.e. WSP complexes C1-57) are identified as groups of proteins sharing three out of four HVR haplotypes (SHVs) with a primary founder (in blue); subgroup founders (yellow) connect to double HVR variants (DHV). In red are recombinant proteins within a complex, labeled with the corresponding allele number or a representative allele in case of multiple variants (*). For clarity only complexes with at least three proteins or recombinants were labeled (C1-19, C47, C51). 183 singletons were removed. Refer to Additional file 2 for profile and allele identification within complexes.

The 515 alleles analyzed correspond to 435 distinct proteins (see additional file 2). Among these, the clustering program eBURST identified 57 complexes of closely related proteins (hereafter named C1 to C57), grouping 251 out of the 435 proteins (Fig. 3). The remaining 184 proteins did not show a clear affiliation to any complex and were excluded from this analysis. The major complexes identified were C1 to C10; complex C1 and C2 were the largest, each comprising 23 distinct profiles/proteins.

Among identified complexes, 29 contained at least three distinct but related proteins for which the putative founder protein could be predicted (Additional file 2). We note that assignment of the founder genotype by eBURST does not take into account the number of allele variants coding for it, nor its genotype frequency across isolates. Nevertheless, within each complex, typically the predicted founder was also the protein with the highest number of allele variants, and the one found in the largest host taxonomical range (see below). These lines of evidence further support the accuracy of the founder sequence assignment. We used these complexes and predicted founders to assign directionality to the evolutionary changes and to estimate relative rates of mutation and recombination.

### Recent evolution of WSP sequences: interplay of mutation and recombination

**Figure 4:**
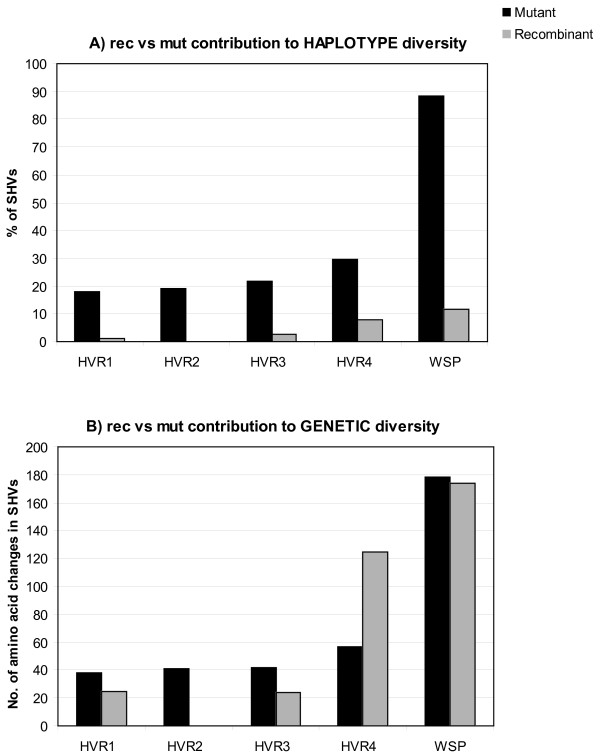
**Relative contribution of recombination (rec) *versus *mutation (mut) to WSP haplotype (A) and genetic (B) diversity**. A) Percentage of recombinant and mutant SHVs per class of HVRs and per protein; B) total number of amino acid changes introduced in SHVs by recombination and mutation per class of HVR and per protein. Overall ratio of rec/mut is about 1/8 to WSP haplotype diversity and about 1/1 to WSP amino acid diversity.

Within complexes, examination of the number of amino acid changes between single hypervariable region variants (SHVs) and the founder protein indicates that the majority of new HVRs arose by point mutation (Fig. 4A), and none by indels. This is true for all four HVRs. Overall, 88.5% of the newly evolved proteins arose by point mutations. Nevertheless recombination was responsible for generating a substantial amount of the new proteins (11.53%, see also Fig. 3 in red). Among HVRs, HVR2 was the least recombinant, with no events detected in our dataset (Fig. 4A). HVR4 showed the highest number of both recombination (7.7%) and mutation (29.5%) events, suggesting it is either a hot spot for both processes or variants in HVR4 are more often selectively advantageous. It should be noted that we are not observing the actual frequencies of recombination and mutation, but rather those that have persisted long enough to be detected. Therefore, these mostly represent variants that are either neutral or selectively advantageous.

In terms of the relative contribution of recombination versus mutation to WSP genetic diversity (Fig. 4B), it is notable that in half of the recombinant cases detected (9/18) recombination introduced more than 10 amino acid changes. As a result, although mutation contributes the most to generating new proteins/HVR peptides (i.e. overall haplotype diversity: 88.46% mutation vs 11.53% recombination, Fig. 4A), the contribution of the two forces to WSP amino acid diversity is almost equal (50.56% vs 49.43%, Fig. 4B). As a striking example, although the relative contribution of mutation and recombination to HVR4 haplotype diversity is approximately 3:1 (Fig 4A), the relative contribution to HVR4 amino acid diversity is 1: 2 (Fig. 4B): 75% of the observed HVR4 amino acid diversity is in fact due to recombination.

When analyses were expanded to include subgroups within complexes (Fig. 3, subgroup founders in yellow), the proportion of recombinants increased from 11.53% to 28.6%: 10 out of 35 subgroup profiles were recombinants at one HVR at least, all 10 showing more than 10 amino acids changes with respect to the ancestral HVR.

### A large impact of recombination among more divergent WSP

Estimating recombination between complexes was more challenging, as without a common founder genotype we could not assign directionality to recombination events. However, the sharing of identical HVRs among distinct complexes with little similarity at the remaining portion of the protein suggests that recombination is also fairly common among more divergent proteins. Among the most common HVRs in the dataset, for example, peptide HVR1-1 is found in 35 distinct proteins (46 alleles) spanning six WSP complexes (Table 2); the average amino acid pairwise divergence among these proteins, excluding HVR1, is 12.27%, suggestive that HVR1-1 was acquired in different *Wolbachia *via recombination. The sharing of the same HVR among otherwise different proteins could also be due to it being an ancestral conserved motif. To investigate this issue, we examined synonymous diversity (*Pi-syn*) of the specific hypervariable motif across all alleles in which it was present and comparing this to that of CRs for the same set of alleles (Table 2). On average *Pi-syn *per hypervariable motif was significantly lower than the average *Pi-syn *values calculated for CRs (average 0.0145 vs.0.0484, Wilcoxon test W+ = 1, W- = 35, N = 8, p < 0.01562). This argues against the hypothesis that common HVRs found in diverse WSP proteins are ancestral and supports, in several cases, their recent shuffling via recombination.

**Table 2:** Most common HVR peptides in the dataset and genetic diversity of proteins and nucleotide alleles in which they are found.

			*Pi-syn*	
Peptide	No. of Prot/alleles in which they are found	Average Prot diversity (%)^1^	HVR^2^	CRs^3^
**HVR1-1**	35/46	12.27	0.017	0.055
**HVR1-2**	23/29	5.45	0.004	0.027
				
**HVR2-12**	24/34	8.93	0.004	0.016
**HVR2-17**	21/28	7.48	0.017	0.041
				
**HVR3-15**	29/33	14.77	0.014	0.082
**HVR3-12**	23/31	4.56	0.013	0.008
				
**HVR4-25**	34/42	13.25	0.041	0.108
**HVR4-23**	29/40	7.64	0.006	0.050

^1 ^excluding the shared peptide

^2 ^average synonymous diversity (*Pi-syn*) of the HVR, excluding the short strings of flanking CRs

^3 ^average *Pi-syn *at the three CRs

### Diversifying selection acting on HVRs

To further investigate whether WSP is target of positive selection during early stages of diversification, we analyzed the selective pressures acting on a) the overall *wsp *sequence during early divergence, and b) single partitions of the gene over longer evolutionary times. For the first approach we identified the ancestral allele from the sets of mutant alleles within each complex. In all but three cases, the ancestor predicted by TCS corresponded to the protein founder identified by eBURST, confirming the above prediction (see Table 3). For the three cases of incongruence, TCS prediction was considered the most reliable because it is based on nucleotide substitutions. We used the ancestral alleles to assign directionality to the nucleotide substitutions and to investigate selection on the alleles. For recently diverged alleles, average dN/dS within complexes ranged from 0.03 to 17.38. The null hypothesis of neutral evolution could not be significantly rejected for 20 out of 25 complexes examined (Tajima'D, P > 0.05), indicating that recently diverged alleles do not typically experience strong selection. To test for positive selection acting at specific sites, we estimated dN/dS values per codon for 29 complexes that each contained at least three sequences. Previously identified allele founders were used for rooting the trees input into codeml and for analysis of the mutational pattern during early allele divergence (within complexes). For the majority of the complexes (17 out of 29) the LRTs between the two model comparisons were not significant. Only five of 29 complexes showed evidence of positive selection at a few sites (see additional file 3). These positively selected sites mostly fall within HVR motifs and involved alleles coding for double hypervariable region variants (DHV) founders and DHVs, suggesting that a signature of positive selection becomes visible with an increase in sequence divergence.

**Table 3:** Predicted founder alleles of major WSP complexes and corresponding profiles, estimated using TCS.

			Founder WSP profile			
Complex	No. of prot/alleles	Ancestral allele^1^	HVR1	HVR2	HVR3	HVR4
**C1**	23/33	wsp-23	1	12	21	19
**C2**	23/31	wsp-11	9	9	12	9
**C3**	15/15	wsp-310	51	143	15	25
**C4**	14/20	wsp-160	2	17	3	23
**C5**	14/21	wsp-61	18	16	23	16
**C6**	13/16	wsp-10	10	8	10	8
**C7**	10/10	wsp-18	13	15	17	14
**C8**	6/7	wsp-238*	104	114	111	99
**C9**	6/10	wsp-91	30	28	31	30
**C10**	6/9	wsp-156	71	34	15	25
**C11**	5/6	wsp-24	17	19	22	18
**C12**	5/5	wsp-28	21	21	25	21
**C13**	4/4	wsp-308	2	142	131	23
**C14**	4/6	wsp-291	24	24	27	26
**C15**	4/5	wsp-33	1	23	15	25
**C16**	4/4	wsp-427	8	1	182	138
**C17**	4/4	wsp-277*	11	13	133	14
**C18**	3/5	wsp-448	112	131	43	114
**C19**	3/5	wsp-64	35	35	38	44
**C20**	3/4	wsp-266	111	130	3	23
**C21**	3/4	wsp-398	71	174	176	25
**C22**	3/3	wsp-195	5	5	94	5
**C23**	3/3	wsp-89	54	28	62	60
**C24**	3/4	wsp-63	19	17	24	33
**C25**	3/5	wsp-54	38	39	46	43
**C26**	3/5	wsp-505	42	43	198	25
**C27**	3/3	wsp-332	136	8	10	132
**C28**	3/9	wsp-138	74	20	24	80
**C29**	3/3	wsp-508*	181	149	147	120

^1 ^P of parsimony of mutational steps >95%

* The corresponding profile did not match eBURST prediction

In the second approach, we considered HVRs and CRs individually and identified sets of closely related sequences within each of the seven sections of WSP, separately (section borders are shown in Fig. 1A, excluding CR1 that was missing in our alignment). We then estimated the average dN/dS for each group within single sections and averaged these values across groups. This global analysis of dN/dS showed that the average dN/dS values for each HVR is typically >1 (see additional file 4), implying diversifying selection. The exception was HVR3, for which the average falls below 1 (0.73) although several groups of related sequences within HVR3 showed dN/dS >>1. In contrast, each CR appears to be under strong purifying selection, with dN/dS<<1 (average range across the three CRs is 0.07 - 0.14). Therefore, when comparing sequences among closely related WSPs (which have only recently diverged) we find only a weak signature of diversifying selection. However, when comparing similar HVR variants that occur in different WSP proteins, we find a strong signature of selection for amino acid substitutions. This finding suggests that those HVR variants that spread via recombination into different WSP proteins can become targets of diversifying selection.

### WSP synonymous variants are evolutionary unstable

We finally tested whether protein genotypes are typically stable over long evolutionary times by investigating the diversity of nucleotide variants coding for a single protein sequence. The rationale is that, if a particular protein genotype is evolutionarily stable over time, it will progressively accumulate synonymous substitutions while discarding amino acid changes. Therefore, the occurrence and genetic diversity of synonymous variants should reflect how long a protein type has been around.

The analyses showed that the majority of WSP proteins exist as a single allele variant in our dataset (91%), with only 8.7% having multiple allele variants (n = 38). Of these proteins, most had only two variants (n = 27), while six proteins had at least four allele variants. Interestingly, each of these six proteins corresponded to the predicted ancestral protein of a complex, confirming their ancestral status. On average WSP proteins are coded by 1.19 allele variants, with an average synonymous diversity (*P*_*syn*_) of 0.27% (Table 4). When compared to any of the five MLST gene datasets, WSP showed the lowest number of allele variants per protein variant and values of *P*_*syn *_among them (Wilcoxon two-sample test P < 0.001 for all comparisons, Table 4). Specifically, the average *P*_*syn *_value among *wsp *variants is at least half that of any of the MLST genes. Such a difference is even more striking considering that the WSP dataset is much larger in terms of allele diversity than any MLST locus dataset.

**Table 4:** Allele diversity per protein type estimated for *wsp *and the five MLST genes

Gene	No. of alleles	No. of prot with multiple allele variants	No. of allele variants per prot (average)	*Pi-syn*^1^
***wsp***	515	38	1.19	0.0027
***gatB***	97	14	1.27	0.0053
***coxA***	76	8	1.49	0.0187
***hcpA***	95	9	1.25	0.0071
***ftsZ***	74	6	1.76	0.0086
***fbpA***	151	20	1.30	0.0056

^1 ^Average allele synonymous diversity per protein

Analysis of the polymorphic pattern within groups containing three or more allele variants per WSP protein type showed, in all cases, that all polymorphisms are unique to a single allele, (i.e. without homoplasies) indicating independent evolution through radiation from the ancestral allele.

### Host population structure of WSP

**Figure 5:**
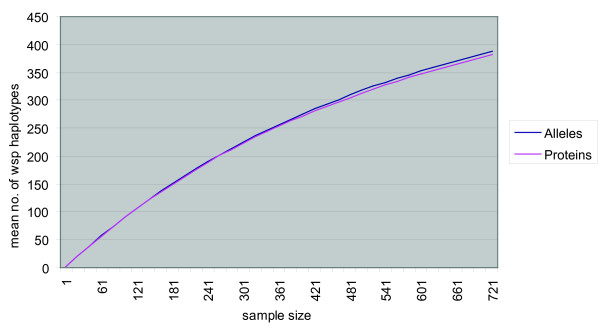
**Rarefaction curve of the cumulative number of distinct *wsp *alleles and proteins**. Analysis is based on a random resampling of 20. The best curve fit was y = -1.347224537·10^-1 ^x + 1179.975114 x/(x + 1034.575827), with a very small residual of rss = 2.251531074·10^-1^. There was no good fit to a curve with asymptotic features.

The 515 *wsp *alleles were found in overall 831 *Wolbachia *isolates, spanning all major arthropod host taxa and continents (see additional files 5 and 1 for a summary and for the whole dataset, respectively). Specifically, the 515 were distributed in 23 orders, 126 families, 296 genera and 475 species. In terms of variety of *wsp *alleles collected, a rarefaction curve indicated that we are still far from saturation of global diversity of this gene (see Fig. 5). Fitting the data to a nonlinear function gave a best fit three-parameter curve that is continually increasing, but with and asymptotic rate of increase of approximately 0.74 new allele variants for every 10 additional sequenced strains, signifying that *wsp *variants in nature are potentially infinite. The allele and protein curves almost perfectly overlap (Fig. 5), supporting the above evidence for shorter persistence of synonymous variants due to accelerated microevolution of the protein (i.e. a persisting new allele is most likely to give rise to a new protein haplotype than to a synonymous variant, leading to similar nucleotide and amino acid diversity).

Based on the global analysis, there is a strong support for a non-random association of WSP with host genus. The results suggest that *wsp *as a gene or associated *Wolbachia *strains are preferentially found in similar hosts. Specifically, the amino acid pairwise distances among WSP sequences found in the same host genus (100 host genera show at least two distinct species in our dataset) are, on average, significantly lower than they would be by random chance (average Pi = 0.1937, P < 0.000). We further explored this association for individual genera focusing on those that were highly represented in our dataset. Results indicated that within most of the genera, the WSP sequences were significantly more similar than expected based on a random sampling (Table 5). Among others, a significant association was found within *Drosophila *hosts (43 entries in the dataset), the spider mites *Tetranychus*, the wasps *Trichogramma *and *Pegoscapus*, and the mosquitos *Culex*. This is the result of either the presence of a single *wsp *allele across species within a genus, as in the case of *Anastrepha*, or more often the presence of several *wsp *alleles sharing high similarity within a genus, as in the case of *Drosophila*, *Trichogramma*, *Tetranychus *and *Culex*.

**Table 5:** Pairwise analysis for significant association (in bold) of similar WSP sequences within the same host genus (1000 replicates).

Genus	No. of Isolates	No. of Host species	No. of *wsp *alleles	P_mean	P_median
***Drosophila***	43	27	27	**<0.000**	**<0.000**
***Trichogramma***	30	16	20	**<0.042**	<0.244
***Tetranychus***	21	6	19	**<0.001**	**<0.005**
***Spalangia***	15	8	14	<0.287	<0.065
***Bactrocera***	14	8	12	<0.087	<0.122
***Pheidole***	13	12	11	<0.414	<0.141
***Solenopsis***	13	4	12	<0.357	<0.246
***Agelenopsis***	13	8	7	<0.321	<0.190
***Armadillidium***	9	4	8	**<0.000**	**<0.000**
***Orius***	8	4	3	**<0.007**	**<0.006**
***Acraea***	7	6	7	**<0.003**	**<0.018**
***Anastrepha***	6	6	1	**<0.000**	**<0.000**
***Synergus***	5	5	3	<0.314	<0.224
***Culex***	8	7	8	**<0.000**	**<0.000**
***Nasonia***	5	4	4	**<0.007**	**<0.008**

**Figure 6:**
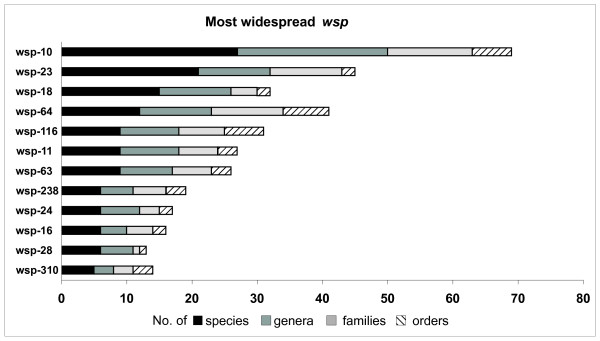
**Most widespread *wsp *alleles and relative host taxonomic distribution**. Note that some of the most common alleles are predicted founders of complexes (e.g. wsp-10, 23 and 18). wsp-10 is the most widespread in terms of diversity of host species, genera and families and corresponds to the ancestral allele of C6 (see also Fig. 3 and Table 3).

We also investigated whether there was any evidence for some *wsp *sequences and thus potential strains to be generalist, while others to have a more restricted range of distribution. We thus looked at the most common *wsp *alleles in the dataset and their distribution among hosts (Fig. 6). Among all alleles, wsp-10 was the most widespread in the dataset, in terms of number of distinct host species (n = 27), genera (23), families (13) and orders (6) in which it was found. Among the six orders, wsp-10 is prevalently found in Lepidoptera and Hymenoptera. Similarly wsp-23 occurs in 21 distinct species, 11 genera, 11 families, although it is restricted to two orders, Diptera and Hymenoptera.

Of the 12 most common alleles in Fig. 6, 10 are predicted founders of WSP complexes (Table 3). Because of the accelerated evolution of this gene, such a broad distribution of a single allele variant appears to be the result of either a recent *wsp *lateral transfer coding for a particularly adaptive WSP haplotype or the rapid host-range expansion of a *Wolbachia *strain associated to such *wsp *sequences, rather than the persistence of an ancestral genotype.

## Discussion

Surface proteins found in intracellular bacteria are extremely interesting candidates for the study of symbiotic interactions, given their location at the interface between the bacteria and the host cell environment. Nevertheless functions and molecular processes driving their evolution in invertebrate hosts have been poorly investigated. Only recently, studies are providing examples of how adaptive immune response in insects can be linked to specific OMP polymorphic variants found in their bacterial symbionts [54], thus unveiling a largely underestimated specificity of the invertebrate immunity [15].

Here we investigated the molecular processes driving diversification of the outer membrane protein WSP, one of the most abundant *Wolbachia *proteins found in *Drosophila *eggs. Based on protein folding prediction, we have shown that WSP presents a typical eight β-barrel structure spanned through the outer membrane and connected to four extracellular loops. The four loops face on the same side of the barrel, and presumably make contact with the host cytoplasm or the vacuole intermembrane space that envelops *Wolbachia*. The lack of strong compositional and conformational constraints at the loops is consistent with their impressive diversification and putative function as receptors, as shown in several OMPs [11,13,14]. On the other hand, the large conservation of the WSP transmembrane core (up to 90% and no indels) likely reflects structural constraints, providing the architectural anchor of the protein to the membrane.

### Microevolution of WSP is largely driven by recombination

WSP shows the most remarkable pattern of recombination seen among the *Wolbachia *proteins studied so far [31]. While recombination involving shuffling of HVR motifs across WSP sequences has been well documented and explains the existence of a remarkable repertoire of WSP protein variants, it remains unclear how this motif diversity is generated and whether it is functional. Here we attempted to reconstruct the microevolutionary steps in WSP early diversification with the aim of uncovering the major forces at the basis of this variation. The dataset used, which comprises 515 alleles found in more than 831 isolates spanning a great taxonomical host range, reflects a substantial sampling diversity of WSP and allowed grouping of recently diverged WSP proteins and HVR motifs.

The reconstruction of WSP relationships presents several technical issues that are common to the analysis of other recombinant OMPs (e.g. the multigene family of P28 OMPs of *Ehrlichia*). In particular there are challenges to detecting true homology via straightforward alignments, due to the high level of sequence and length variation, and issues relating to correcting for the recombination bias while attempting to detect and measure selection acting on the molecule [4,55]. Indeed, most WSP and other OMPs-based studies have excluded the HVRs to avoid alignment issues, although this resulted in exclusion of crucial functional domains of the protein. On the other hand, studies that have relied on global alignments have faced extreme alignment problems, making assumptions on true homology difficult or impossible. Here we approached these issues using a profile-based method for proteins grouping, which does not base on alignments. This clustering method allowed the identification of several sets of recently diverged WSP proteins and their ancestral genotypes, thus providing statistical power to investigate sequence evolution on relative short-time scales. Specifically, by grouping similar haplotypes at HVRs we were able to discriminate between amino acid changes introduced by mutational versus recombinational events and analyze the contribution of the two forces to the actual protein (thus functional) diversity, and not to allele diversity. Results indicated that while mutation in WSP occurred at a higher frequency than recombination, as expected, recombination has had a remarkable impact both on the emergence of novel protein types and on the very rapid increase of genetic diversity among proteins, being responsible alone for about 50% of the total amino acid variation observed among recently diverged proteins. Shuffling of WSP portions among more divergent sequences is also frequent, as indicated by the sharing of identical HVRs among otherwise very divergent alleles. Such a pattern strongly suggests that recombination is ongoing and largely contributed to both the short- and long-term diversification and evolution of WSP.

How did this WSP mosaicism generate? A similar pattern of diversification is observed in other OMPs of vertebrate pathogens, such as in *Neisseria *Opa proteins [12,56] and MSP2 of *Anaplasma *[57]. Variability in these proteins is typically generated via a process of gene conversion involving modular cassettes of HVR motifs located in pseudogenes within the same genome [56,57]. Unlike these proteins, *wsp *occurs as single copy in the genome, based on all published *Wolbachia *genomes and evidence from PCR amplifications using universal *wsp *primers, which typically return clear single sequences. We searched for presence of additional single HVR motifs in the published *Wolbachia *genomes from *Drosophila melanogaster *and *Culex pipiens*, but failed to detect any significant homology to full or partial HVR motifs (data not shown). This suggests that WSP chimeric structure is primarily due to recombination events involving foreign DNA rather than a modular exchange of hypervariable regions within a single genome. Modes of DNA transfer across *Wolbachia *strains remain unknown, although the widespread occurrence of coinfections of a single host clearly provides a suitable arena for DNA exchange.

### Loop diversity is adaptive

The predicted four extracellular loops, which accommodate the HVR motifs, show an extreme plasticity and a mutational pattern that appears largely unpredictable. Indels, which represent one of the major sources of diversity among WSP sequences and only occur at HVRs, were absent among recently diverged proteins (i.e. in SHVs) but numerous among recombinants, suggesting that they are normally introduced via recombination. Among the four loops, L4 presents the largest variation, due to both mutation and recombination. There is no apparent restriction in L4 size, which can vary by as much as 19 amino acids in length. This loop versatility does not disrupt the reading frame, which was always conserved, and thus strongly suggests that this diversification is adaptive. In contrast, L3 showed the lowest variation in length (two amino acids difference) and the lowest dN/dS ratio, which could be due to larger structural constraints or simply to a lower recombination impact. Regardless of length plasticity, it is, however, interesting to note that all four loops show similar haplotype diversity (Table 1), with a potential to accommodate a very large repertoire of distinct peptides. Nevertheless, some compositional constraints are expected: 1) AT-biased codons are strongly favored, as indicated by the high AT content at third (synonymous only) codon positions of *wsp *(83%) as well as of *Wolbachia *housekeeping genes (76%), and 2) amino acid composition should account for a high percentage of hydrophilic amino acids, given that these sites are extracellularly exposed.

What are the types of selective pressures acting during the early diversification of WSP? Alleles appear to be under neutrality during recent divergence, although we cannot exclude statistical limits in detecting positive selection in very closely related sequences. A signature of selection becomes visible with increasing sequence divergence. HVRs and CRs are clearly evolving under very different selective pressures, as shown by average dN/dS values typically >1 for HVRs, and <<1 at the CRs. Although average dN/dS values for some HVRs approximate 1, thus suggesting neutrality, we note that these dN/dS values are averaged across codon sites, as well as across groups of sequences within single HVRs. On the other hand, CRs are clear targets of strong purifying selection, likely due to structural constraints.

Concordant with a large impact of recombination and diversifying selection, allele diversity per protein type indicates that WSP is a highly unstable protein. WSP shows the lowest number of synonymous variants per protein type and the lowest synonymous diversity when compared to any of the five MLST housekeeping genes, despite the use of a much larger dataset for WSP. This suggests that any single protein haplotype does not persist for long periods of time and that selection for amino acid diversification is likely ongoing. Indeed, all nucleotide substitutions observed among synonymous allele variants are unique, with no homoplasic events, supporting their recent and independent divergence. Similarly, identical HVRs motifs found in distinct proteins typically show a very low synonymous divergence, which suggests that either they have recombined quite recently, or more likely that HVR synonymous variants are particularly unstable after settling into a new allele and are soon target of nonsynonymous substitutions. This implies that those HVR variants that spread via recombination into different WSP proteins can rapidly become targets of diversifying selection and are thus adaptive to some extent. Evidence that OMP genetic diversity can play a crucial role in host-symbiont interaction comes from the native symbiont *Sodalis *of the tsetse fly *Glossina morsitans*, where polymorphisms at the exposed loop of the outer membrane protein OmpA were shown to mediate host tolerance, determining the host/symbiont type of interaction (pathogenic and not) [54]. We speculate that the extensive variation at WSP extracellular loops could also play a similar role in escaping or down-regulating the immune system by means of rapid turnover of exposed amino acids.

### Population structure of WSP

Previous studies have shown that WSP-based relationships are typically incongruent with inferences based on other *Wolbachia *housekeeping genes, suggesting that WSP is often horizontally transferred as a single gene, uncoupled from the rest of the genome [58]. While the same or similar *wsp *alleles can often occur in otherwise very divergent strains, and therefore reconstructions of strain relationships based solely on *wsp *should not be trusted, on average closely related host taxa tended to harbor strains with significantly closer WSP sequences than observed between strains from more phylogenetically distant hosts. This appears the result of a preferential transfer of the entire *Wolbachia *strain among closely related hosts (or codivergence of strains) rather than WSP alone, as supported by previous studies using MLST data [59,60]. However, because *wsp *represents the only genetic information for the majority of isolates included in this study, the two scenarios cannot be discriminated at this time.

Despite an overall non-random association of WSP with the host genus, few *wsp *haplotypes (e.g. wsp-23 and wsp-10) were widespread across a large host taxonomical range. It is noteworthy that *wsp*-23 and 10 alleles are typically found in two of the most widespread *Wolbachia *strains identified by MLST (ST-13 and ST-19 respectively, [57,61]), suggesting that *wsp *distribution in this case largely reflects the distribution of these two *Wolbachia *strains.

## Conclusions

Large recombination impact, diversifying selection, lack of strong compositional and structural constraints in WSP extracellular loops, and frequent horizontal gene transfer are signature features of adaptive evolution. These features are typically found in proteins targeted by the adaptive immune system (such as OMPs of vertebrate pathogens) [3,13]; however, *Wolbachia *infect invertebrates only. There is growing interest in understanding whether and how *Wolbachia *escape or down-regulate the host immune responses so that they can exist within host cells. By combining the structural analysis with the microevolutionary analysis of WSP, we can speculate that the extracellular loops contain peptide motifs that serve to evade or inhibit host detection, aiding in the early settlement and persistence of *Wolbachia *into a new host. Biochemical investigations exploring binding properties of WSP are currently ongoing and will help elucidating the role of WSP in the invertebrate hosts.

## Authors' contributions

LB conceived of the study, carried out the molecular genetic analyses, and drafted the manuscript. JHW participated in the design of the study and helped to draft the manuscript. CAD provided the statistical tool for the sequence-host association analysis. JAR and JKS contributed in the acquisition of data and provided comments to the manuscript. All authors read and approved the final manuscript.

## Supplementary Material

Additional file 1 The 831 *Wolbachia *isolates studied. Click here for file

Additional file 2 WSP profiles of the 515 alleles. Click here for file

Additional file 3 Positive selected sites within WSP complexes, as detected by codeml. Click here for file

Additional file 4 **dN/dS values estimated for hvrs and CRs of WSP**. The graph shows differential rates of evolution along the gene. Each value was averaged across dN/dS values calculated for groups of related sequences at each of the seven sections of WSP (see Fig. 1A). Groups of sequences were defined by 100% matching length and at least 95% of nucleotide identity. Click here for file

Additional file 5 **Relative frequency of *wsp *sequences per host country (A) and taxonomic order (B)**. All major arthropod orders and continents are represented. NAM: North America; EUR: Europe; AFR: Africa; NEAS: North East Asia; CAM: Central America; SAM: South America; AUS: Australia; PAC: Pacific Ocean Islands; SEAS: South East Asia; CAS: Central Asia. Click here for file
