# Supplementary material for: Accelerated microevolution in an outer membrane protein (OMP) of the intracellular bacteria Wolbachia
Source: BMC Evol Biol. 2010 Feb 17;10:48. doi: 10.1186/1471-2148-10-48 (PMC2843615; doi:10.1186/1471-2148-10-48)
Supplement: Additional file 3 — Positive selected sites within WSP complexes, as detected by codeml. [file 1471-2148-10-48-S3.PDF]

Complex	Best models[1]	Positive selected sites[2]
C1	M2, M8	**7 T *, 8 Y **, 12 K****, **59 P***
C3	M2, M8	**56 K ****
C4	M2, M8	**10 T **, 11 G***
C6	M2, M8	135 F**, **152 A***
C7	M2, M8	30 G*

[1] Best models resulting from LRT of two comparisons M1 vs M2 and M7 vs M8. Chi square cutoff significance was set to P<0.01.

[2] Amino acid reference sequences correspond to the ancestral allele of each complex (see Table 3). In bold are sites falling within HVRs.

*P>95%, **P>99%
