# Supplementary figures and images for: Accelerated microevolution in an outer membrane protein (OMP) of the intracellular bacteria Wolbachia
Source: BMC Evol Biol. 2010 Feb 17;10:48. doi: 10.1186/1471-2148-10-48 (PMC2843615; doi:10.1186/1471-2148-10-48)

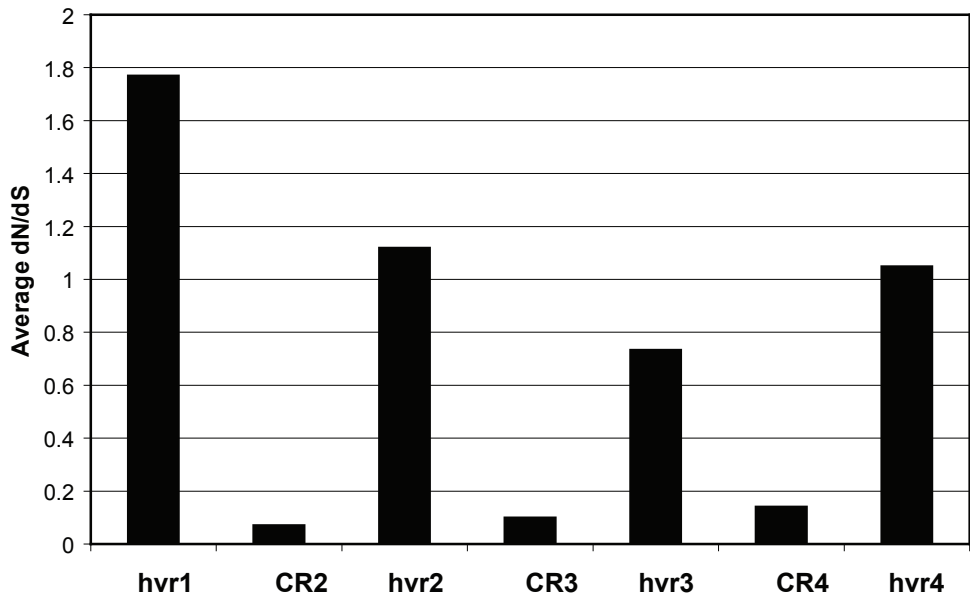

Supplement: Additional file 4 — dN/dS values estimated for hvrs and CRs of WSP. The graph shows differential rates of evolution along the gene. Each value was averaged across dN/dS values calculated for groups of related sequences at each of the seven sections of WSP (see Fig. 1A). Groups of sequences were defined by 100% matching length and at least 95% of nucleotide identity. [file 1471-2148-10-48-S4.PDF]

## A) Frequency of Host countries

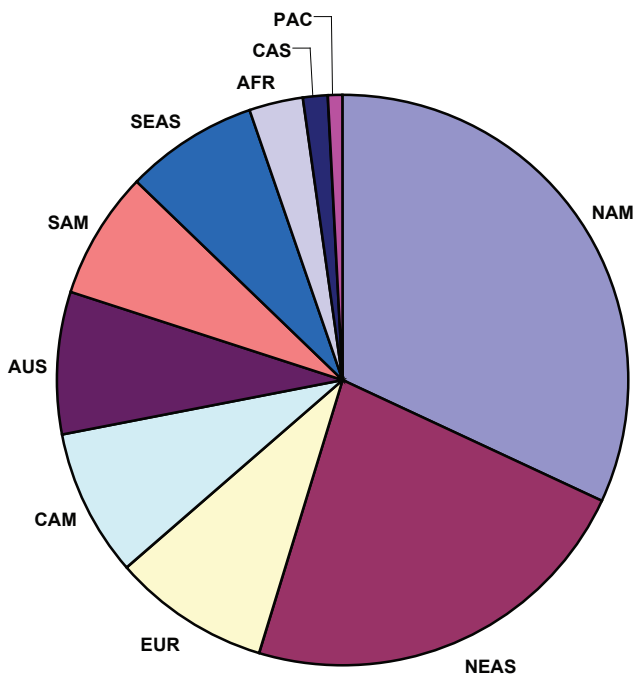

## B) Frequency of Host orders

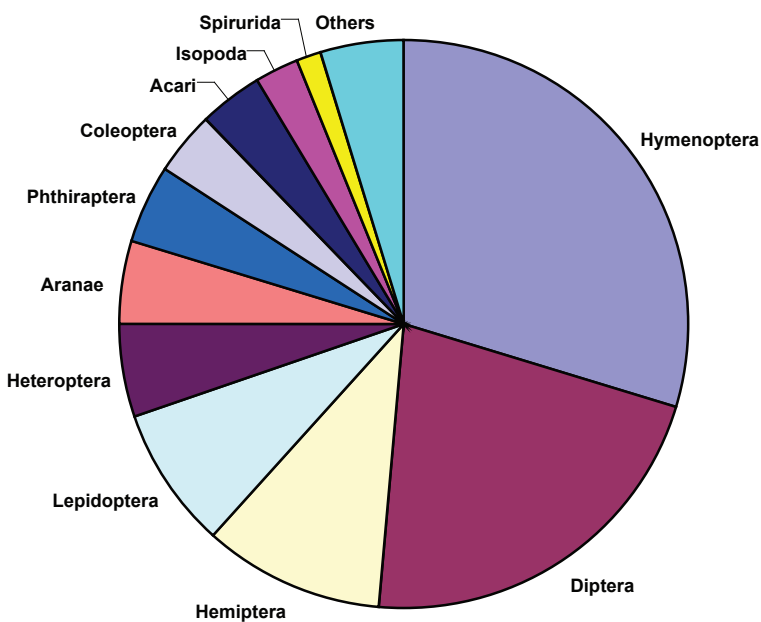

Supplement: Additional file 5 — Relative frequency of wsp sequences per host country (A) and taxonomic order (B). All major arthropod orders and continents are represented. NAM: North America; EUR: Europe; AFR: Africa; NEAS: North East Asia; CAM: Central America; SAM: South America; AUS: Australia; PAC: Pacific Ocean Islands; SEAS: South East Asia; CAS: Central Asia. [file 1471-2148-10-48-S5.PDF]
